# Synthesis of nano-hydroxyapatite using emulsion, pyrolysis, combustion, and sonochemical methods and biogenic sources: a review

**DOI:** 10.1039/d3ra07559a

**Published:** 2024-01-22

**Authors:** Md. Kawcher Alam, Md. Sahadat Hossain, Md. Kawsar, Newaz Mohammed Bahadur, Samina Ahmed

**Affiliations:** a Glass Research Division, Institute of Glass & Ceramic Research and Testing, Bangladesh Council of Scientific and Industrial Research (BCSIR) Dhaka-1205 Bangladesh shanta_samina@yahoo.com; b Department of Applied Chemistry and Chemical Engineering, Noakhali Science and Technology University Noakhali Bangladesh; c BCSIR Dhaka Laboratories, Bangladesh Council of Scientific and Industrial Research (BCSIR) Dhaka-1205 Bangladesh

## Abstract

Hydroxyapatite (HAp) is comparable to materials in bone because its chemical components are similar to those contained in animal bone, and thus, its bioactive and biocompatible properties are similar. There are applications for HAp and relevant calcium phosphate in the medical and industrial sectors, and due to the rising demand for HAp nanoparticles, considerable work has been performed to develop a variety of synthetic pathways that incorporate scientifically and practically novel aspects. Numerous studies have been conducted to examine how changes in reaction parameters will successfully influence crucial HAp features. HAp can also be synthesized from biogenic sources such as HAp-rich fish scales or animal bones as an alternative to chemical precursors. Various preparation techniques produce crystals with varying sizes, but it has been found that nano-sized HAp exhibits a greater number of bioactive properties as compared to micron-sized HAp. Rather than considering conventional methods, this review focuses on alternative approaches such as emulsion, pyrolysis, combustion, and sonochemical methods along with waste bio-sources (biogenic sources) to obtain HAp. We summarize the currently accessible information pertaining to each synthesis process, while also focusing on their benefits and drawbacks.

## Abstract 2

Different synthesis methods (emulsion, pyrolysis, combustion, and sonochemical) of calcium phosphates and exploration of biogenic sources.

## Graphical abstract

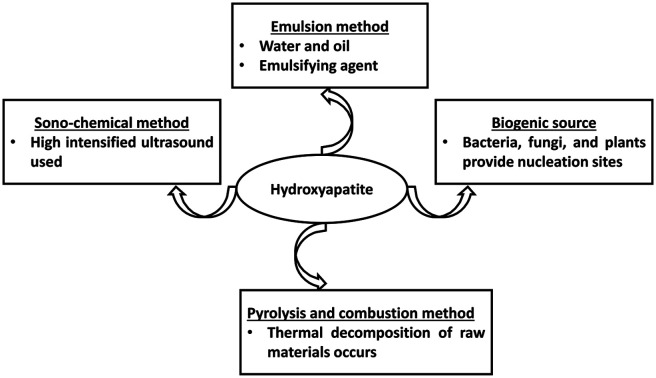

## 1 Introduction

Hydroxyapatite (HAp) is a synthetic bioactive substance used in cutting-edge hard tissue engineering due to its strong chemical connection with bone tissue, and it is employed as a reliable bone transplant material.^1^ The bioactivity of HAp facilitates bone regeneration, and its chemical composition is comparable to the mineral component of genuine bone.^2–4^ Naturally occurring CaP is typically HAp that is carbonated and calcium-deficient, with a Ca/P ratio of less than 1.67.^5,6^ Due to its great osteogenic potential, superior biocompatibility, and affinity for biopolymers, considerable research has been conducted on synthetic HAp for many years.^7–11^

The mineral component of bone is most closely related to the CaP salt hydroxyapatite (Ca_10_(PO_4_)_6_(OH)_2_), which is the most thermodynamically stable crystalline form of CaP in bodily fluid.^12,13^ Among the numerous HAp forms, research has been conducted on nanosized HAp, also known as HAp nanoparticles (NPs) with proper shapes.^14^ It is generally established that bioceramics with composition and structure similar to those of bone minerals can more easily stimulate osteointegration and subsequent bone tissue growth.^5^ According to reports, nanosized HAp-based ceramic biomaterials are substantially more bioactive and resorbable than micron-sized ceramics.^15–17^ With nanosized HAp, researchers can more easily comprehend the mechanism and create more optimal biomedical devices, such as implant coverings,^18^ bone scaffolds,^19^ and bone fillers.^20,21^

Due to HAp's significance in tissue rejuvenation and as a drug carrier, numerous techniques for creating HAp NPs have been documented. For HAp to be effectively used in biological applications, particle size and shape are two critical considerations.^22^ While HAp production and particle size have been well-researched, there are surprisingly few studies that discuss how HAp is controlled by its morphology.^23^ The methods mainly used for the synthesis of HAp NPs include solid state,^24^ mechanochemical,^25^ chemical,^26^ hydrolysis,^27^ sol–gel,^28^ pyrolysis,^29^ combustion,^30^ sonochemical,^31^ and emulsion.^32^

All of these techniques are used for the synthesis of HAp with different particle sizes and morphologies. To modify the Ca/P ratio of 1.67 for prepared HAp, the preferred approach is that of wet mechano-chemical, and if this ratio is not maintained, then the process will yield β-tricalcium phosphate (TCP) as the second phase.^33^ HAp can also be obtained from biogenic sources, such the bones of animals and fish scales, as an alternative to chemical synthesis. However, these require extensive chemical treatment to remove organic compounds or unwanted parts.

Researchers found that the bones of most animals contain nanosized HAp, which possesses an increased capacity for bio-resorbability as compared to micron-sized HAp, and therefore, there has been considerable interest in developing nanosized HAp.^34,35^ In comparison to micron-sized HAp, nanosized HAp exhibits greater bioactivity and biocompatibility. Similar to biological apatite, nanosized HAp releases calcium ions similarly and much more quickly as compared to larger crystals.^36,37^ Additionally, nano-sized HAp enhances cellular activity and cell proliferation during bone formation, and therefore, it is an ideal biomaterial for bone implants.

**Fig. 1:**
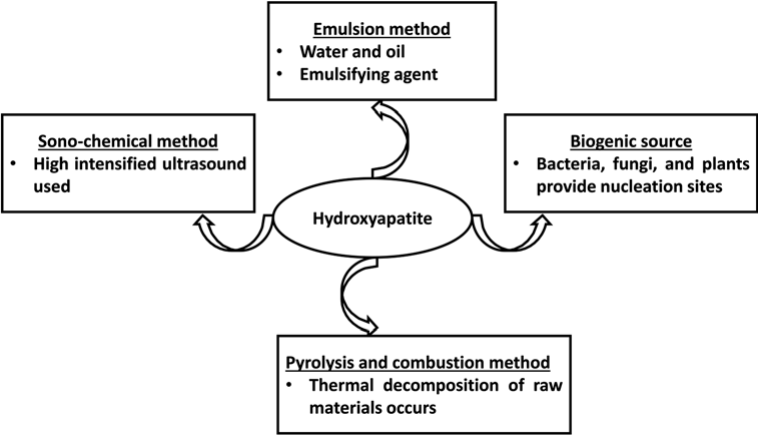
Schematic representation of the diverse techniques employed in the synthesis of nano-HAp.

To create a formation of HAp that is similar to the HAp in animal bone and teeth, many characteristics have been explored.^38^ Synthetic bone implantation offers enormous prospects for medical care, and regeneration of bone defects has evolved to be a common transplant procedure, with blood transfusions being the most common.^39,40^ This review focuses on the emulsion, combustion, pyrolysis, and sonochemical methods of HAp synthesis. Biogenic sources are also discussed, in addition to conventional synthesis processes (Fig. 1).

The emulsion method involves creating a stable emulsion of water and oil, emulsified with an agent, and introducing HAp precursors, which will result in the formation of nano-HAp particles within the emulsion system.^41^ The thermal decomposition of organic precursors, either by pyrolysis or combustion, produces nano-HAp. Pyrolysis occurs without oxygen, while combustion occurs with oxygen, resulting in nanostructured HAp.^42^

Bacteria, fungi, or plants are biological organisms that can be used as biogenic sources that function as templates or nucleation sites to synthesize nano-HAp, resulting in the generation of nanostructured particles.^43^ Sonochemical methods induce acoustic cavitation in a liquid medium *via* high-intensity ultrasound, which leads to the development of nanostructured HAp particles, facilitated by the presence of localized heated regions and elevated pressure.^44^

## 2 Methods of synthesis for HAp NPs

Numerous methods are used to synthesize synthetic HAp: solid state,^24^ mechanochemical,^25^ chemical,^26^ hydrolysis,^27^ sol–gel,^28^ pyrolysis,^29^ combustion,^30^ sonochemical,^31^ and emulsion.^32^ Another widely available source of HAp is bio-resources derived from biogenic sources.^45^ Although this is not a synthesis method, bio-resources are very important for safe applications in different biomedical fields. In addition to producing distinct crystalline phases of HAp and β-TCP, each of these processes produces HAp with varied sizes and morphologies.^38^ To determine the distinctions and complications of each approach in the preparation of HAp crystals, each synthesis method was evaluated.

### 2.1 Emulsion method

**Fig. 2:**
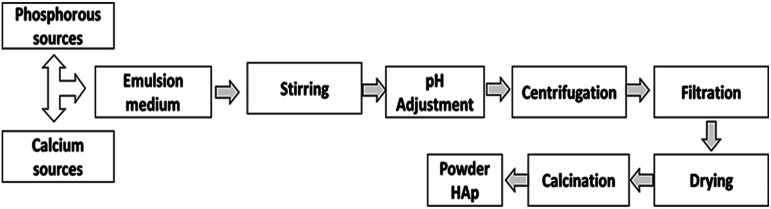
Synthesis of HAp using the emulsion method.

In the emulsion system (Fig. 2), two or more immiscible liquids consisting of a disperse phase and continuous phase, which is generally oil and water where liquid droplets are dispersed in a liquid medium.^46^ The emulsion can be W/O or O/W based on the types of disperse and continuous phases. When oil is distributed into water, then the resulting emulsion is called ‘oil in water,’ but in the case of ‘water in oil,’ the roles of oil and water are reversed.^47–50^

Micro- or macro-emulsions can also be used, depending on the extent of the incorporated water. A macro-emulsion contains a relatively higher volume of water than a micro-emulsion.^51,52^ The reaction mechanism of the emulsion method depends on the collision of water droplets. When two water droplets come in contact, the reaction is completed. HAp is formed using an emulsion technique that yields uniform particle size with a large surface area.^53–55^ In an emulsion approach, one of the precursors is initially combined with the emulsion medium, and the reaction is then continuously stirred until the necessary Ca/P adjustment is made. Finally, the finished product can be separated from the emulsion medium using a demulsifier or centrifugation.^56^

HAp has been synthesized from Ca(OH)_2_ and H_3_PO_4_, with the use of glycerin as the emulsion medium. The resultant particles were calcined at 600–800 °C to confirm the thermal stability at different phases. If the reaction temperature and pH are maintained at 37 °C and 10, respectively, the final product will be a long-lasting apatite phase. However, a Ca/P ratio lower than 1.67 and pH below 7.4 will form TCP or dicalcium phosphate dihydrate (DCPD).^57^ Kimura *et al.* synthesized a multiple emulsion (w/o/w) to produce microsized HAp crystals using dipotassium hydrogen phosphate [K_2_HPO_4_] solution, benzene, and Ca(NO_3_)_2_·4H_2_O as the water, oil, and water phases, respectively. X-ray diffraction (XRD) patterns ensured that the synthesized products were composed of porous microspheres smaller than 3 μm in size.

For a full day at 323 K, a multilayered emulsion experiment was conducted. One-phase HAp was manufactured at an initial pH level of 12, and the crystalline phase was changed according to the initial pH of the internal water phase. The product consisted of porous microspheres less than 3 μm in size, and the nanospheres that composed the microspheres were smaller than 120 nm in size. The main advantage of this technique was that the product can be obtained at low reaction temperatures.^48,54^ Mesoporous HAp was prepared from Ca(NO_3_)_2_·4H_2_O and H_3_PO_4_ using a double emulsion technique according to the following reaction:^58^

1 10Ca(NO_3_)_2_·4H_2_O + 6H_3_PO_4_ + 20NH_4_OH → Ca_10_ (PO_4_)_6_(OH)_2_ + 20NH_4_NO_3_ + 22H_2_O

A freeze-drying process was developed to synthesize macroporous HAp granules using camphene as a porogen material.^59^ Gonda *et al.* developed an alternative emulsion technique to synthesize microporous HAp granules, although these granules were not suitable for tissue engineering applications because of their small internal pores.^60^ The effect of reaction temperature and Ca/P ratio on the properties of HAp particles was also monitored when surfactants were used as the emulsion medium, and CaCl_2_·2H_2_O and (NH_4_)_2_HPO_4_ were used as the reactants. A calcium-deficient amorphous phase final product was obtained, and it was observed that high calcination temperatures reduced the surface area of the particles. It was also found that a Ca/P ratio of 2.00 encouraged low crystallinity and the formation of TCP.^61^

Another biomaterial with biocompatibility and bioactivity similar to that of HAp is β-TCP. β-TCP and α-TCP can be obtained along with HAp as the second and third phase. If the reaction Ca/P ratio is maintained at less than 1.67 and the pH is lower than 7.4 in a glycerol/water emulsion, the apatite phase breaks down into β-TCP. A weak crystalline apatite structure was observed for synthetic calcium phosphates, and they appeared to be a nearly pure β-TCP phase when the temperature was elevated to 800 °C. Different phases of HAp, TCP, or biphase calcium phosphate (BCP, which contains HAp and TCP) can be obtained by altering the pH value, Ca/P ratio, and adding glycerol.^57^

Somnuk *et al.* discovered that producing HAp particles at a high Ca/P molar ratio of 2.00 caused a sizable amount of β-TCP to be produced. There was no impact of the initial Ca/P molar ratio modification on particle size.^61^ Metal-doped HAp NPs can also be prepared by the emulsion technique. Chen *et al.* developed an emulsion approach that was used to prepare gold nanorod (GNR)-doped HAp microspheres, and their microstructure and photo-thermal properties were examined. Nano-emulsification was used to create single-phase Sr-substituted HAp that can be used to immobilize Sr radioactive isotopes. According to certain reports, adding strontium to HAp widens the crystal size distribution.^62^ A few examples are registered in Table 1.

**Table 1:** Synthesis of HAp using emulsion method from different Ca and P sources with relevant properties

Ca source	Ca(OH)_2_	CaCl_2_·2H_2_O	Ca(NO_3_)_2_·4H_2_O	Ca(NO_3_)_2_·4H_2_O	Ca(NO_3_)_2_·4H_2_O
P source	H_3_PO_4_	(NH_4_)_2_HPO_4_	(NH_4_)_2_HPO4	K_2_HPO_4_	(NH_4_)_2_HPO_4_
Reaction temperature (°C)	37	30–80	25–30	100	25–30
Reaction pH	10	—	7–11	9–12	—
Morphology	Rod shape	Spherical	Spherical/nanorod	Porous microspheres	Short rod shape
Amorphous/crystalline	Crystal	Amorphous	Crystal	Crystal	Crystal
Size from XRD (nm)	800–1300	—	—	—	—
Size from TEM^a^ (nm)	—	Less than 70	Rod: *L*: 200–280	100	*D*: 10–30
			*W*: 20–25		
			Spherical: *D*: 20–35		
Size from SEM	0.5–3 μm	Less than 70 nm	—	3 μm	—
Sintering temperature (°C)	1300	450–750	—	—	600
References	63	64	50	65	66

a *L* = length, *W* = width, and *D* = diameter.

The results in Table 1 indicate that the synthesized HAp reached an amorphous phase when CaCl_2_·2H_2_O was utilized as the Ca source. The morphology of the generated HAp was also influenced by the sintering temperature, because a high temperature produces a rod-shaped crystal.

### 2.2 Pyrolysis method

**Fig. 3:**
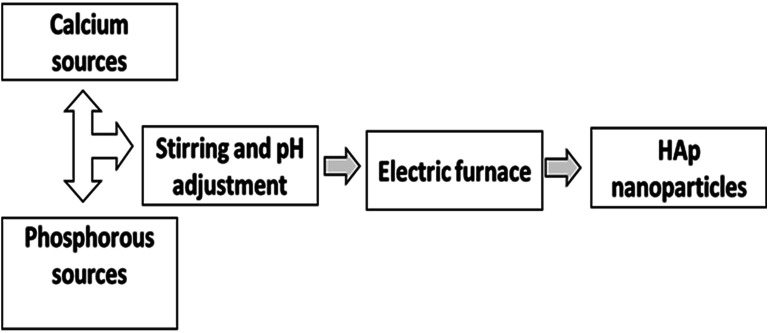
Synthesis of HAp using the pyrolysis method.

The pyrolysis method is a technique where a precursor chemical is sprayed into the hot zone of an electric furnace.^21,67^ Particles then form through an aerosol method, whereby they are generated by the conversion of a liquid/gas into a particle.^68,69^ It also involves the generation of gas vapors, because the reaction of the initial solution as it is sprayed into a flame using an ultrasonic generator and high temperature is responsible for the formation of NPs.^70,71^ The final product is found in an aggregated form due to the spray pyrolysis process. The difference between the pyrolysis and combustion method is that the pyrolysis technique does not use any type of fuel that is mixed with the reactant solution.^72,73^ The pyrolysis method is visualized in Fig. 3.

Using the spray pyrolysis method with calcium phosphate, calcium nitrate tetrahydrate, and diammonium hydrogen phosphate as the precursors, HAp phases were obtained. Reactants were mixed at 1100 °C with a carrier gas to produce the final powder. Field emission scanning electron microscopy (FESEM) characterization indicated that the HAp powder was composed of micron-sized spheres.^74^ Spray pyrolysis was also used to synthesize HAp with a combination of calcium acetate and diammonium hydrogen phosphate, and the atomised reactant solution was heated at 500–1000 °C. At the lowest temperature, the dominant HAp phase was not present, according to the XRD pattern.^75^

A method of pyrolysis was used that employed a high-temperature flame spray to produce nano-sized HAp crystals, with preliminary reactants of calcium nitrate (Ca(NO_3_)_2_·4H_2_O) and di-ammonium hydrogen phosphate ((NH_4_)_2_HPO_4_). After post-treatment at 600–1000 °C, the morphology and mean diameters of the HAp crystals produced from the polyethylene glycol (PEG) spray solution ranged from rod-shaped to spherical and were 32–213 nm in size, respectively. It was discovered that while non-spherical forms and aggregated structures were found in the powders synthesized from PEG solution, spherical shapes and micron-sized crystals were produced when PEG solution was not used.^76^

Another technique for producing HAp crystals smaller than one micron is known as drip pyrolysis on a fluidized bed. Using the drip pyrolysis method, Nakazato *et al.* conducted the reaction at 640–840 °C. According to the data from an SEM study, at 640 °C, most of the particles were greater than 40 mm in size, and at 840 °C, the majority of the particles were less than 10 mm in size.^77^ The spray pyrolysis technique is limited because it can only produce HAp with large surface areas, and therefore, salt-assisted decomposition is used to overcome this limitation. Salt-assisted decomposition of spray pyrolysis adds a salt, NaNO_3_, to force NP formation, and a final product was obtained after removing the salts. When salt was added in concentrations ranging from 1 to 50 weight percent, the shape of the particles changed from sphere to rod, and 5 weight percent of NaNO_3_ caused a reversible decrease in particle size.^78^

Using a dewaxing technique with a solution of calcium phosphate and PEG, nano-sized HAp crystals were produced. When a calcium phosphate solution containing PEG was sprayed at 1100 °C, loosely packed HAp crystals smaller than a micron in size were created. However, when dewaxed at 650 °C, the crystals were entirely reduced to nano size, whereas micron-sized carbon-free particles were left intact. The average particle size range of the specimens was 1.94 ± 0.71 μm.^79^

When Ca(N0_3_)_2_ and (NH_4_)2HPO_4_ were used as the initial reactants in the spray pyrolysis method, β-TCP was discovered as the second phase. The resulting powder's crystalline phases were revealed by the XRD pattern to be HAp and a small amount of β-TCP, although it is challenging to discern their overlapped reflections because of the low crystallinities and the presence of β-TCP reflections nearby. When the temperature in the upper furnace was lower than 850 °C, β-TCP was found in the powders. When the higher furnace temperature was increased to 900 °C, only HAp was present, and raising the upper and lower furnace temperatures improved the crystallinity of the resulting HAp powder.^80^ A few examples appear in Table 2.

**Table 2:** Synthesis of HAp using pyrolysis method from different Ca and P sources with relevant properties

Ca source	Ca(NO_3_)_2_·4H_2_O	Ca(NO_3_)_2_·4H_2_O	Ca(NO_3_)_2_·4H_2_O	Ca(NO_3_)_2_
P source	H_3_PO_4_	(NH_4_)_2_HPO_4_	(NH_4_)_2_HPO_4_	(NH_4_)_2_HPO_4_
Reaction temperature (°C)	700	—	200–400	640–840
Reaction pH	—	1.5	—	—
Morphology	Rod shape	Hollow spherical	Rod shape	Flat shape
Amorphous/crystalline	Crystal	Crystal	Crystal	Crystal
Size from XRD	—	—	42 nm	—
Size from TEM (nm)	20–40	35–100	213	—
Size from SEM	200 nm	1.94 μm	—	10–40 μm
Sintering temperature (°C)	—	1100	1000	940
References	81	82	83	84

The results shown in Table 2 suggest that when phosphoric acid (H_3_PO_4_) was used as the phosphate source, the final product was formed as nanocrystallite rod-shaped HAp. This indicates that the morphology and crystallite size rely on the phosphate source of the precursors.

### 2.3 Combustion method

An exothermic reaction in an aqueous medium between an organic fuel such as glycine or urea and a suitable oxidant is the combustion technique used for the production of HAp.^85–87^ The solution combustion method is suitable for producing HAp with excellent mechanical properties because the particles formed are denser and nanosized.^88^ In the combustion synthesis process, the raw components are each separately dissolved in a solvent before being combined and heated in a furnace. Agglomerates of particles that can be broken down by attrition are obtained as the final result.^89^

Due to the exothermic nature of the process, the reaction can begin at nearly room temperature without the need for additional heat.^30,90,91^ Because temperature rises in direct proportion to reaction duration, high reaction temperatures are ideal for separating undesirable contaminants. The particle size of the final product is very small because rapid cooling prevented the particles from growing.^92^ Using glycine and urea as organic fuels during combustion, calcium nitrate and diammonium hydrogen orthophosphate were converted into HAp. For the urea and glycine system, the combustion temperature was found to be 896 °C and 1035 °C. The types of powders formed depend on the composition of the fuel, and a lack of fuel at low flame temperature resulted in HAp with a high surface area, but powders with weak porous agglomerates were produced in the fuel-rich reaction.

It was noted from the SEM micrograph that as the fuel-to-oxidizer ratio rises, the proportion of porosity or void volume also increases.^93^ Microwave-assisted combustion is a perfect candidate for HAp synthesis because it offers high crystallinity at low temperatures. Heat is produced inside the molecule due to the successive transfer of radiation. The XRD result for microwave-assisted HAp crystals showed a sharp peak at 600 °C after calcination.^94^ HAp can also be synthesized from eggshells by employing citric acid as the combustion fuel, and HAp was found to be the predominant phase with satisfactory crystallinity. The absence of an XRD peak at 37.36° confirmed the absence of CaO.^95^ HAp was also produced with CaO by maintaining the combustion temperature at 500 °C for 15 min and using calcium nitrate and dihydrogen ammonium phosphate as the reactants and urea as the combustion fuel, and the XRD pattern indicated the presence of CaO with the main HAp phase.^96^

Kavita *et al.* used different chemical reagents and combustion times to synthesize pure crystalline HAp, using calcium acetate and diammonium phosphate as the main chemical precursors.^97^ A modified combustion synthesis method was used to create calcium phosphate-based bioceramics by employing succinic acid and citric acid alone and in combination as fuels.^98^ According to powder X-ray diffraction (PXRD), citric acid or succinic acid alone will produce the HAp phase, while a combination of citric acid and succinic acid will produce the β-TCP phase.^99^ The temperature at which HAp breaks down into TCP and calcium oxide was observed to be reduced when the carbonate content of the HAp lattice increased.

The carbonated apatite formed by mixed fuel completely transformed into β-tricalcium phosphate at 900 °C, which may suggest that there is a greater carbonate component.^100,101^ Hong *et al.* mentioned that the surface area of metal containing HAp decreased with increasing combustion temperature.^102^ However, Venkatachari *et al.* supported the opposite outcome, which was that due to the rapid reaction, the surface area of zirconia powder increased as the furnace temperature increased in metal nitratesoxalic dihydrazide aqueous combustion.^103^ An X-ray diffractogram showed that CaHPO_4_ is produced when NH_4_OH is employed as a basic solvent and EDTA is used as a complexing agent at a reaction temperature of 60 °C. However, at calcination temperatures, CaHPO_4_ can fuse with the production of β-TCP. At 800 °C, the pure phase powder is produced, and it is stable up to 1200 °C. When the material is further calcined at 1300 °C, there is negligible conversion to α-TCP.^104^ A few examples are listed in Table 3.

**Table 3:** Synthesis of HAp using combustion method from different Ca and P sources with relevant properties

Ca source	Eggshell	Ca(NO_3_)_2_·4H_2_O	Ca(NO_3_)_2_·4H_2_O	Ca(NO_3_)_2_	Ca(NO_3_)_2_
P source	(NH_4_)_2_HPO_4_	(NH_4_)_2_HPO_4_	(NH_4_)_2_HPO_4_	(NH_4_)H_2_PO_4_	(NH_4_)H_2_PO_4_
Reaction temperature (°C)	70	500	300–700	500	600
Reaction pH	9.5	—	—	7.4	3.5
Morphology	Spherical	Spherical	Porous foam-like shape	Rectangular	Isometric spherical
Amorphous/crystalline	Crystal	Crystal	Crystal	Crystal	Crystal
Size from XRD (nm)	44 in diameter	—	17–18	33	—
Size from SEM	—	5–200 μm	31–50 nm	—	70–100 nm
Size from DLS	—	—	—	—	—
Sintering temperature (°C)	900	1230	900–1040	—	1000–1200
References	95	89	93	30	92

**Fig. 4:**
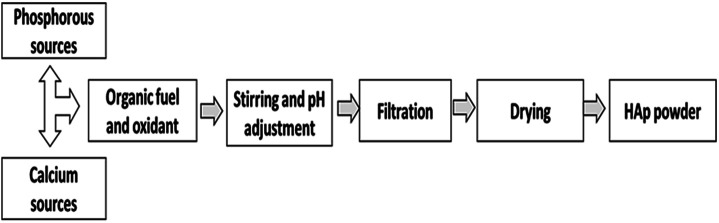
Synthesis of HAp using the combustion method.

The data in Table 3 show that the size of the produced crystal is significantly influenced by the reaction temperature and pH of the reaction process. High reaction temperatures and low pH values result in smaller HAp nanocrystals. The combustion method is presented here in Fig. 4.

### 2.4 Sonochemical method

Sonochemistry is the study of molecules that undergo chemical reactions as a result of the use of strong ultrasonic radiation (20 kHz to 10 MHz). Because the generated particles are small in size, with great uniformity and sphericity, and a greater crystalline percentage and specific area with low agglomeration, sonochemical methods are used in HAp synthesis.^105,106^ In the case of sonochemical synthesis, the precursor solutions are first combined while being continuously stirred. The reaction medium is then placed into a program-controlled microwave reactor with sonochemistry assistance. The medium is typically maintained between 50 and 80 °C, and a high-intensity ultrasonic probe is used to adjust the frequency.^31,107,108^

Varadarajan *et al.* found that increased crystal aspect ratios with needle-like morphologies resulted from ultrasonication, which can increase the degree of nano-crystallinity in HAp that is lacking in calcium.^106,109^ HAp was produced from Ca(NO_3_)_2_ and (NH_4_)_2_HPO_4_ using the sonochemical approach at a frequency of 20 kHz. The XRD pattern of synthesized HAp shows that the crystals are small and highly pure. All samples, however, showed four faint β-TCP peaks, indicating the presence of contaminants in the synthesised HAp. The nanoplate-like morphology of the synthesized HAp particles, which have sizes ranging from 8 to 50 nm, was validated by TEM images. Interestingly, with a sonication time of 20 min, there were clearly smaller diameters (8 nm) and reduced powder agglomeration rates.^108^

Using a homogeneous precipitation method in an ultrasound field, nanosized platelike HAp was synthesized. The synthesized HAp nanorods were approximately 500 nm long with a diameter of approximately 100 nm. The resulting diffractogram demonstrated that the sonochemically synthesized HAp is monophase, and there were no other diffraction maxima except the maxima that corresponded to the HAp structure. A comparison of sonochemical precipitation and ordinary homogeneous precipitation without ultrasound shows that the sonochemical method yields monophase HAp with controlled morphology and crystal size.^110^ Through an expedited sonochemical procedure, calcium-deficient hydroxyapatite (CDHAp) NPs with a Ca/P ratio of 1.6 were created.

Calcium nitrate and diammonium hydrogen phosphate were used in the synthesis, which was carried out in an ultrasonic bath with a fixed frequency of 135 kHz and 250 W power. It was noted that as the reaction temperature increased during precipitation, the size of the crystallites also increased. However, it has been shown that when precipitation occurs as a result of ultrasonication, the crystallite size increases for the first 60 minutes of ultrasonication before beginning to decrease for the next 60 minutes, and then increases once more. Without ultrasonication, non-homogeneous morphologies were observed in the TEM micrograph for the particles, whereas uniform rods for 90 minutes and needle-like protrusions for 120 minutes of ultrasonication were observed.^109^

Phosphoric acid and calcium carbonate particles derived from eggshells were used in the sonochemical production of bio-based HAp NPs. In this process, calcium and phosphorus were obtained from eggshell particles and phosphoric acid solution, respectively. The produced HAp particles were crystalline, porous, and thermally stable to at least 750 °C, according to the TEM data.^111^ The general reactions are presented as eqn (2)–(4):

2 CaCO_3_ → CaO + CO_2_↑

3 CaO + H_2_O → Ca(OH)_2_

4 5Ca(OH)_2_ + 3H_3_PO_4_ → ½Ca_10_(PO_4_)(OH)_2_ + 9H_2_O

Using a microwave-assisted sonochemistry technique, carbonated HAp nanopowders were created. The effects of microwave and ultrasonic irradiation on the crystallinity, morphology, Ca/P molar ratio, specific surface area, and dispersibility were investigated and contrasted with the traditional precipitation methods. In approximately 5 minutes, well-crystallized nanopowders were produced with a high yield of 98.8%. Rod-like HAp crystallites with a diameter of approximately 8 nm and a length of approximately 30 nm were successfully dispersed during simultaneous microwave and ultrasonic irradiation.^112^

There were four tiny peaks of β-TCP for all samples when calcium nitrate and di-ammonium hydrogen phosphate were used as the primary precursors with natural latex rubber, indicating the presence of contaminants in the synthesized HAp. Variations in the Ca/P ratio due to the development of complexes with trace elements present in natural latex rubber led to the formation of an impurity phase. The major HAp phase did not significantly vary at different ultrasonic irradiating times. HAp and β-TCP phases were mixed together in the XRD pattern. In the absence of natural latex rubber, the pattern thus unequivocally supports the existence of a mixed phase of non-stoichiometric HAp.^113^ A few examples appear in Table 4.

**Table 4:** Synthesis of HAp using sonochemical method from different Ca and P sources with relevant properties

Ca source	Ca(NO_3_)_2_·4H_2_O	Ca(NO_3_)_2_·4H_2_O	Ca_3_(NO_4_)_2_·4H_2_O	Eggshell	Ca(NO_3_)_2_
P source	(NH_4_)_2_HPO_4_	(NH_4_)H_2_PO_4_	(NH_4_)_2_HPO_4_	H_3_PO_4_	(NH_4_)_2_HPO_4_
Reaction temperature (°C)	25	88	—	25–30	80
Reaction pH	—	—	10	—	10.8
Morphology	Nanoplate-like shape	Rod-like shape	Needle-like shape	Needle-like shape	Rod-like shape
Amorphous/crystalline	Crystal	Crystal	Crystal	Crystal	Crystal
Size from XRD (nm)	36–44	Diameter 100	24–26	—	29
		Length 500			
Size from TEM (nm)	8–50 diameter	0.34–0.47	50	30–50	8 in diameter
					30 in length
Sintering temperature (°C)	1200	—	700	750	—
References	108	110	114	111	112

**Fig. 5:**
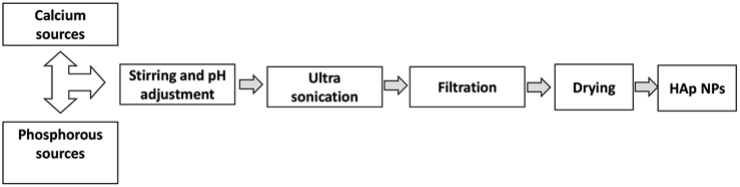
Synthesis of HAp using the sonochemical method.

The reaction temperature influences the morphology of the final product, as the data indicated that a reaction temperature at approximately 80 °C will produce rod-shaped HAp. The sonochemical process is shown in Fig. 5.

## 3 Biogenic source/bio-source derived

Biogenic sources are satisfactory alternatives for synthesizing biocompatible and bioactive biomaterials for use within the human body because of their unique features.^115–117^ It is a priority for today's researchers to develop methods for the proper utilization of waste materials so that they can be transformed into effective products. HAp can be synthesized from natural and synthetic sources, and natural materials are the most beneficial and cost-effective.^118^ Eggshells, fish bones, and mussel shells are potential sources of raw materials for the preparation of HAp.^119,120^ Eggshells are composed of calcite, whereas the cuttlefish bone is made of aragonite, but mussel shells are composed of a combination of calcite and aragonite.^121,122^

Fish scales, fish bones, and bones from cows and pigs are animal waste materials that have been utilized to obtain HAp. One technique that has been used to recover HAp from bio-waste is simple heat degradation. A straightforward technique called thermal breakdown is used to create HAp from pig bones. Bone sludge, or deproteinized and defatted bone pulp, is the basic material. Bone sludge was subjected to a two-stage calcination process to prepare HAp.^45^ Using physical, chemical, and thermal procedures, animal bones are converted into desired biogenic HAp (BHAp). The nonstoichiometric crystalline structure of BHAp, which results from the replacement of ions, is one of its most alluring characteristics.^116^

Biogenic calcium carbonates are very useful for the preparation of biocompatible HAp. Most of the HAp produced from biogenic origin is calcium deficient or contains metal ions as impurities, such as Mg in eggshell-derived HAp.^123^ Amorphous calcium carbonate can also be derived from different types of ocean fishes.^124^ HAp was produced by heating fish bones at 1000 °C under normal atmospheric pressure. The formed flakes were ball-milled at 300 rpm to obtain the desired particle size.^115,125^ In Zaria, Nigeria, HAp was prepared from the waste bones of animals and catfish. After cleaned bones were sintered at 900 °C, the attained powders were introduced into a sieve.^126^ Ferro *et al.* described that the conversion of eggshells into HAp required more energy as compared to the conversion from cuttlefish bones because the reaction mechanisms are totally different for both of these materials.^127^

Rodica *et al.* obtained rod-shaped HAp from eggshells and di-base ammonium phosphate [(NH_4_)_2_HPO_4_] using microwave radiation for the perfect growth of crystals. The processing temperature was maintained at a maximum of 200 °C to inhibit the formation of β-TCP, and the highest calcium percentage was 96.38 wt% in pre-calcined samples.^128^ Brushite and ammonium calcium phosphate monohydrate were observed as the intermediate phases from XRD patterns when the reaction occurred at room temperature. However, nearly pure HAp was found when the sample was subjected to oven-drying temperatures. The XRD peaks also indicated that a drying temperature of 100–150 °C favoured crystal formation.^121^

Satisfactory thermal durability was obtained from HAp synthesized from bovine bone, whereas thermal instability was observed and residues of tricalcium phosphate (TCP) HAp were produced from caprine and galline bones following heat treatment over 700 °C. The porous nature of the bone samples was supported by the discovered microstructures and low relative density. Bovine and caprine bones heated to specific temperatures produced a porous HAp body with hardness values that are similar to those of human cortical bones. For the generation of a fully formed crystalline HAp phase, the optimal calcination temperatures for galline and caprine bones were determined to be 700 °C and 750 °C, respectively.^129^ A few examples are shown in Table 5.

**Table 5:** Synthesis of HAp using biogenic resources from different Ca and P sources with relevant properties

Ca source	Eggshells	Snail shells	Eggshells	Eggshell	Cockle shells
P source	H_3_PO_4_	(NH_4_)_2_HPO_4_	(NH_4_)_2_HPO_4_	H_3_PO_4_	KH_2_PO_4_
Reaction temperature (°C)	—	40	30–120	80	—
Reaction pH	8.8–9.0	—	8.5	9–11	—
Morphology	—	Needle-like/rod-like shape	Flake-like	—	Needle-like shape
Amorphous/crystalline	Crystal	Crystal	Crystal	Crystal	Crystal
Size from XRD	—	12–17 nm	100 μm	20.25 nm	—
Size from SEM	—	—	2 μm	30–80 nm	—
Sintering temperature (°C)	1100–1300	—	975	—	—
References	155	156	121	157	122

Depending on whether it is produced from natural sources or synthetic compounds, HAp can exhibit various morphologies. Apatite crystals created in a biological system differ from crystals produced using various methods requiring synthetic precursors.^130^ Because of their larger surface area and lower crystallite size, the apatite crystals produced in living systems can absorb a greater number of ions. Because of the presence of trace amounts of metal ions, the HAp prepared from mammalian bone is a non-stoichiometric material.^131^ For the purpose of creating scaffolds for directed bone regeneration, the cortical portion of femoral bones is used. It is already well established that the organic portion of a bone enables regulation of crystallite size, size distribution, and lattice orientation during biomineralization processes and also aids in regulating the thickness of apatite crystals.^132,133^

Bio-waste, mainly the bones of cattle, has been used to extract natural HAp. Thermal decomposition, subcritical water, and alkaline hydrothermal processes have all been used to prepare natural HAp. Given that the resulting HAp particles assume a nanorod form and an average length of 300 nm, thermal decomposition results in improved morphology.^134^ To obtain weakly crystalline porous HAp with a mean crystal size of 12 nm, bovine bones were chemically treated to remove unwanted organic materials before being sintered overnight at 500 °C.^135^ After being calcined at 950 °C, phase-pure crystalline HAp was created, although it contained a variety of trace components.

Subcritical water extraction of collagen at 275 °C and basic hydrothermal hydrolysis of the organic matrix at 250 °C are the two different methods used to extract the pure crystalline phase of HAp. However, the easiest method that can be used to obtain pure HAp is thermal degradation of collagen and other organic matter at 750 °C.^134^ The sintering temperature significantly affects the physical and chemical characteristics of HAp synthesized from bovine bone, and it has been observed that 1000 °C is not favourable for the formation of HAp crystals because the crystallinity increases with the sintering temperature from 600 to 900 °C, and HAp degrades into other substances at temperatures greater than 1000 °C.^136^ According to a chemical examination of the filtrate from sample washing, CaO was formed as a secondary phase as a result of the calcination process at 1200 °C.

Nanosized amorphous HAp was produced from bovine bone that was reacted with Ca(NO_3_)_2_ when the bone was burned in an open environment after being ball-milled and basified to pH 10 .^137^ Nanosized erratically shaped HAp was found when camel and horse bones were sintered at 700 °C for two hours. The crystal size of HAp synthesized from camel bones was 97 nm, and 28 nm for horse bones. The findings from this synthesis process show that for sintering crystalline HAp from camel and horse bones, the Ca/P ratios should be 2.036 and 2.131, respectively.^138^ Pig bone was used to produce pure crystalline HAp with a rod-like morphology. Prior to pre-treatment, clean and dried pig bones were calcined at 600, 800, and 1000 °C. The Ca/P ratio of a sample after 1000 °C calcination was 1.88.^139,140^

Another method used for producing HAp from pig bone waste employed thermal annealing at temperatures between 600 and 1000 °C. The microscopic structure of the as-synthesised HAp was determined by SEM and TEM examination to be a rod-like morphology with a length of 38–52 nm. As a result, bio-waste such as pig bones can be used to create porous HAp scaffolds instead of using the customary, traditional chemical technique.^141^ Waste pigeon bones were heated to 850 °C before being cold-pressed into NPs and then re-sintered at 850, 950, 1050, and 1150 °C to create naturally produced nano-HAp. The average particle size of the pigeon-derived nano-HAp produced in a ball mill ranged from 50 to 250 nm.^142^

Around the world, several million tons of eggshells are produced as bio-waste. To stop the spread of dangerous infections into the environment that could harm human health, eggshells must be properly managed as a hazardous waste. Eggshells are an efficient natural source that can be used for HAp production, and therefore, it was encouraged to explore the use of eggshells as a reliable source of calcium in order to support the value-added notion with a sustainable and regenerative element.^143^ Wet chemical precipitation was used to create HAp, utilizing calcium oxide from eggshells along with phosphoric acid. It was calculated that the molar ratio of calcium oxide to phosphoric acid was 2 : 1. For six hours, the reaction was run at 120 °C. The calcination temperature was 800 °C to obtain the greatest beneficial effects on the particles and their homogeneity.^144^ DCPD and eggshell powders were combined, and then ball-milled and heated to create HAp powder. The milled sample was sintered at 1000 °C for 1 hour to initiate the creation of the HAp phase, and the milled sample was then sintered at 1000 °C for 10 hours to produce HAp in the pure phase. Additionally, the final products composed of biphasic calcium phosphate were produced simply by ball-milling for 5 hours and then heating for 1 hour at 1000 °C.^145^

A broad range of solid-state techniques have been used for the large-scale synthesis of HAp from eggshells. However, the main disadvantage of these technique is the emergence of secondary phases, such as β-TCP, which evolved because of its irregular phase properties.^146,147^ Utilizing eggshell waste and H_3_PO_4_ as precursors, Lee *et al.* successfully manufactured pure HAp and β-TCP. To transform CaCO_3_ into CaO, cleaned eggshells were initially sintered at 900 °C for this study.^148^

The entire procedure can be described by the following reactions:

5 CaCO_3_ → CaO + CO_2_

6 2CaO + H_3_PO_4_ → CaHPO_4_ + Ca(OH)_2_

7 5CaHPO_4_ + 5Ca(OH)_2_ + H_3_PO_4_ → Ca_10_(PO_4_)_6_(OH)_2_ + 8H_2_O

Homogeneous mixing of the primary raw reagents with suitable thermal treatment of eggshells are key factors in producing pure HAp from the direct conversion of CaCO_3_. A single-phase HAp with an average crystal size of 54.6 nm was produced by applying the following procedure. The organic components were entirely removed from the eggshell powder by initial calcination at 700 °C for two hours. After being further calcined at 800 °C, the wet attrition milled eggshell powder was combined with DCPD^149^ in a process represented by the following reaction:

8 6CaHPO_4_·2H_2_O + 4CaCO_3_ → Ca_10_(PO_4_)_6_(OH)_2_ + 4CO_2_ +4H_2_O

The consumption of fish and crustaceans leads to the accumulation of huge amounts of Ca- and HAp-rich feces. Calcium, phosphate, and carbonate are abundant in fish bones, which can be utilized to produce HAp. As a result, several beneficial chemicals have been created using marine fish waste. Fish bones or comparable sources are cleaned with hot water, steam, or various alkaline solutions to remove proteins and other organic contaminants before the conversion into HAp.^150^ To produce HAp, the bones are heated to a high temperature during calcination after the protein mass has been removed.

Bigeye tuna (*Thunnus obesus*) bones underwent alkaline hydrolysis with NaOH and were then thermally calcined at 900 °C for 5 hours to release carbonated HAp. When compared to HAp produced from other sources, such as pig or bovine bones, tuna bone HAp was more thermally stable up to 1200 °C.^151^ Pure HAp was prepared from the scales of nilotica fish using an alkaline heat-treatment process. Fish scales that had been thoroughly cleaned and dried were deproteinized and then boiled with 50% sodium hydroxide at 100 °C for one hour to produce HAp.^152^ To create pure HAp scaffolds, Rocha *et al.* hydrothermally transformed fresh cuttlefish bones into HAp.^153^ Shi *et al.* also used a hydrothermal procedure to produce HAp microspheres from cuttlefish bones.^154^

**Fig. 6:**
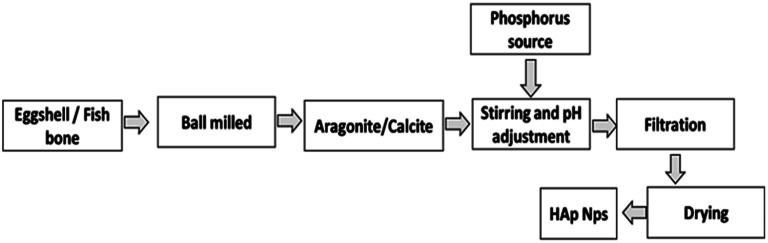
Synthesis of HAp using biogenic resources.

The ultimate output of the synthesis process is flawless nanocrystals, which are produced when eggshells and phosphoric acid are used as sources of Ca and P, respectively. Conversely, HAp that was extracted from snail shells likewise produced perfectly formed nanosized crystals. The utilization of biogenic sources for calcium phosphate synthesis is presented in Fig. 6.

## 4 Conclusion

To obtain crystalline HAp, the different approaches require different processing variables such as pH, Ca/P ratio, reaction temperature, sintering temperature, and reaction time. A balanced Ca/P molar ratio and reaction pH must be maintained according to the desired procedure because these are the most crucial parameters. Chemical modifiers, such as pyrolysis, emulsion, combustion, and sonochemical, are used in some processes to accelerate the reaction. Thus, homogeneous HAp crystals of various sizes and morphologies can be produced.

The most optimal processes for creating HAp with a wide surface area and ideal crystal size are pyrolysis and combustion. Even though the emulsion technique is less expensive than some other processes, it is nonetheless difficult to perform because the finished product must be separated from the medium. There are other options, such as obtaining HAp from a natural source, that can bypass the issues associated with producing synthetic HAp. Fish scales and animal bones can serve as a suitable source of HAp because they include all the beneficial components needed to improve the biological effects of HAp.

## Author contributions

Md. Kawcher Alam collected the data, and wrote the draft and original manuscript. Md. Sahadat Hossain conceived and designed the review, analysed the data, and assisted in writing the manuscript. Md. Kawsar assisted in collecting the data. Newaz Mohammed Bahadur and Samina Ahmed supervised the findings of this work. Samina Ahmed supervised the overall work and managed the required facilities.

## Conflicts of interest

There are no conflicts to declare.

## Supplementary Material
